# Facts about Fats: New Insights into the Role of Lipids in Metabolism, Disease and Therapy

**DOI:** 10.3390/ijms21186651

**Published:** 2020-09-11

**Authors:** Marco Segatto, Valentina Pallottini

**Affiliations:** 1 Department of Biosciences and Territory, University of Molise, Contrada Fonte Lappone, 86090 Pesche (Is), Italy; marco.segatto@unimol.it; 2 Department of Science, University Roma Tre, Viale Marconi 446, 00146 Rome, Italy

**Keywords:** Cholesterol, Fatty acids, Lipid mediators, Lipids, Lipophagy, Sphingolipids

Although initially regarded as a passive system to store energy, lipids are now considered to play crucial, structural and functional roles in almost all the biological processes involved in the regulation of physiological and pathological conditions. For instance, they are pivotal constituents of cell membranes, where they essentially contribute to the assembly of the bilayer configuration. Lipid species are not uniformly distributed in cell membranes, as they are mainly concentrated in specialized sphingolipid- and cholesterol-rich domains called lipid rafts and caveolae, which serve as a matrix for the attachment and the interaction of several proteins implicated in membrane-initiating signal transduction pathways [1]. Besides the involvement in the organization of membrane domains, lipids may influence numerous cellular processes by directly participating as both primary and secondary messengers. In recent decades, lipids derived from both dietary sources and endogenous biosynthesis gained considerable clinical relevance for their implications in a plethora of human diseases. Hyperlipidemia often results in a premature and increased risk of cardiovascular diseases [2]. Increasing evidence highlights that the metabolic reprogramming of cancer cells also involves many lipid compounds, such as fatty acids and cholesterol, which contribute to key oncogenic functions [3,4]. Furthermore, a variety of experimental and clinical studies demonstrated that lipids, particularly cholesterol, play essential roles in brain physiology [5]. Thus, it is not surprising that numerous neurological and neurodegenerative disorders are often accompanied by misbalances in lipid homeostasis [6]. 

Despite a satisfying level of knowledge being reached in the field of lipid research, several questions still remain unanswered; a deeper comprehension will be important to fully elucidate the molecular mechanisms regulating lipid homeostasis in health and loss of homeostasis in disease, and to design innovative therapeutic strategies. This Special Issue, entitled “Emerging Role of Lipids in Metabolism and Disease”, comprises a collection of seven research articles, seven review articles and one concept paper reporting new insights into the biological role of lipids. 

Lipid homeostasis is guaranteed by a delicate equilibrium among biosynthesis, uptake and catabolism. Kloska and colleagues [7] systematically reviewed the current knowledge on lipophagy and cytosolic lipolysis, two selective lipid catabolic processes whose activity is essential for the proper regulation of energy homeostasis in cells. The authors provide an in-depth discussion of the transcriptional modulation of these pathways by the mammalian target of rapamycin complex 1 (mTORC1) and by the transcription factor EB (TFEB). In addition, alterations of both lipophagy and lipolysis in pathological conditions are also documented. Even though basic molecular pathways underlying the maintenance of lipid homeostasis are now well established, several regulatory mechanisms still remain unknown. In this context, the experimental work provided by Tonini and colleagues report a novel epigenetic pathway by which cells physiologically control lipid homeostasis [8]. Notably, the authors unravel that bromodomain and extraterminal domain (BET) proteins, which are epigenetic readers particularly committed to the regulation of cell cycle progression, govern lipid metabolism by modulating the expression of proteins and enzymes implicated in fatty acid and cholesterol biosynthesis, trafficking and uptake. Coherently, BET protein blockade by the small inhibitor JQ1 strongly reduces the amount of intracellular lipids. In addition, Tonini and colleagues also showed that the decreased proliferation of HepG2 cancer cells induced by JQ1 is dependent on the modulation of cholesterol metabolism. The connection between lipid metabolism and the biology of cancer cells is also investigated by Avril et al., who studied the impact of epinephrine-infiltrated adipose tissue (AT) on MCF-7 breast cancer cells [9]. They observed that epinephrine-infiltrated AT secretes different factors, including lipids, which may act as signaling molecules. Indeed, both epinephrine-infiltrated AT and its corresponding conditioned medium (CM) enhance the proliferation of MCF-7 breast cancer cells in vitro. The secreted factors contained in CM also appear to increment the in vivo growth of MCF7 cells in mice. However, injection of whole epinephrine-infiltrated AT did not induce any change in the progression of MCF-7- tumor in mice, suggesting that the employment of CM to mimic the secretome of cells or tissues may explain some divergences observed among in vitro, pre-clinical and clinical data using AT samples. 

Besides cancer biology, alterations of lipid metabolism are a major public health concern because of their association to cardiovascular diseases. Atherosclerosis, a complex process characterized by progressive inflammation and build-up of cholesterol and other lipids in the artery walls, is the leading cause of cardiovascular diseases. The exact etiopathogenesis of atherosclerosis is not fully elucidated, however, several risk factors have been identified, such as high blood pressure, hypercholesterolemia, obesity and diabetes. In their review article, Poznyak and colleagues provided up-to-date information about the use of knockout mice as experimental models to investigate genes involved in atherosclerosis and dyslipidemia [10]. In addition, Poznyak et al. discuss the current knowledge concerning the mechanisms by which diabetes mellitus promotes the atherogenic process, and summarize the physiopathological hallmarks linking atherosclerosis and diabetes mellitus, such as protein kinase signaling, oxidative stress, miRNA alterations and epigenetic changes [11]. The breakdown of homeostatic regulation of lipids occurring during diabetes has also been examined by Primer and collaborators [12]. In particular, they summarize the current understanding of endothelial cell metabolism and its dysregulation during diabetes and discuss the different mechanisms by which high-density lipoproteins (HDL) modulate endothelial cell metabolic reprogramming and counteract diabetes-impaired angiogenesis. 

Lipid metabolism is also finely regulated within the brain: it maintains neuronal functionality and signals the nutrient status to regulate the whole-body metabolism by modulating key peripheral organs and tissues, such as the liver and adipose tissue. Alterations of lipid metabolism have been frequently associated with disturbances in brain functioning, such as neurodegeneration, neuroinflammation, cognitive alterations and neurodevelopmental problems. It is becoming increasingly clear that high levels of dietary fats and sugars, which are typically comprised in a western diet, may be detrimental to brain health. Mazzoli and colleagues provided an experimental work aimed at analyzing the putative effects of a western diet on the metabolic response of nervous and adipose tissue [13]. For instance, they reported that the expression of specific cyto/adipokines, such as TNFα and adiponectin, are significantly affected in both brain and adipose tissue of rats fed with a diet high in saturated fats and fructose (HFF). The observed changes are accompanied by a reduction in brain-derived neurotrophic factor (BDNF) and synaptotagmin I levels, and by an increase in the expression of the post-synaptic density protein, PSD95, in HFF-fed animals. When evaluated as a whole, these results underline that a western diet may induce similar metabolic alterations in adipose tissue and brain. Chun and Chung further confirmed the involvement of dietary lipids in brain physiology [14]. Specifically, they show experimental evidence that a high-cholesterol diet significantly decreases the expression levels of phospholipase C β1 (PLCβ1) and of phosphatidylinositol 4,5-bisphosphate (PIP_2_). Interestingly, there is no direct correlation between the amount of cholesterol and of PIP_2_, suggesting that PIP_2_ levels are modulated by cholesterol through changes in the expression of PLCβ1. Since the reduction in PIP_2_ levels has been associated with β-amyloid production, these results indicate that a high cholesterol diet may influence brain cholesterol, which reflects in PIP_2_ changes that could contribute to the pathogenesis of neurodegenerative conditions. Considering that the blood brain barrier prevents the uptake of lipoprotein-bound cholesterol from the bloodstream, it will be a stimulating challenge to comprehend how diet-derived cholesterol can influence neuronal processes.

Among lipids, sphingolipids represent a major subcategory. These molecules are not only structural components, but also act as bioactive compounds in the mediation of physiological processes involved in cell proliferation, survival, inflammation, senescence and death. A number of experimental evidence sustains that the metabolic pathway, which governs sphingolipid metabolism, exerts a pivotal role in the regulation of the response to DNA damage. Francis and colleagues analytically reviewed how sphingolipid signaling influences the DNA damage response (DDR) induced by metabolic stress, ionizing radiation or other genotoxic stimuli [15]. In particular, they illustrate how different sphingolipid metabolites interact with the mediators of DDR to define cell fate. In the field of sphingolipid research, Cataldi and colleagues provided new interesting insights about the involvement of sphingomyelinases in the effects of ionizing radiations. Notably, experimental data highlighted that ionizing radiations cause altered hepatic cell structure and increased caspase-1 expression in mice. These effects are attenuated by the administration of recombinant manganese superoxide dismutase (rMnSOD). Importantly, rMnSOD counteracts the radiation-induced liver damage exerting a protective role via acid sphingomyelinase (aSMase), and a preventive role via neutral sphingomyelinase (nSMase) [16]. Cataldi et al. also demonstrated that nSMase is responsible for the preventive and protective effect elicited by rMnSOD against radiation-induced damage in the brain [17].

As already mentioned above, lipids not only serve as structural components, but also exert crucial biological roles as signaling molecules. This aspect is extensively discussed in the review article proposed by Kloska and collaborators, who focused their attention on the role of lipid mediators in the risk and pathology of ischemic stroke [18]. Notably, a lively metabolism of polyunsaturated fatty acid has been documented in ischemic brain, and different lipid mediators are implicated in the neuroprotective or neurodegenerative effects occurring in the post-stroke brain tissue. Among signaling molecules, eicosanoids seem to play crucial roles in the disease pathology, and a variety of reports suggest them as useful molecular targets for innovative therapeutic interventions. The involvement of lipid molecules as signaling mediators is further examined in the comprehensive review by Suryadevara et al., which collects up-to-date knowledge about the pathways mediated by lipid mediators in pulmonary fibrosis [19]. Indeed, the metabolism of phospholipids, sphingolipids, and polyunsaturated fatty acids may generate key molecules capable of signaling properties, which exhibit pro- and anti-fibrotic effects in patients and preclinical models of idiopathic pulmonary fibrosis (IPF). In light of this evidence, it is not surprising that prostanoids, lysophospholipids, sphingolipids and their metabolizing enzymes are currently under active investigation as potential pharmacological targets to treat IPF.

Finally, Carbone and collaborators contributed to this Special Issue with a research article aimed at assessing the association of circulating C-reactive protein (CRP) levels with epicardial and visceral fat depots in women with one or more defining criteria for metabolic syndrome [20]. The main findings highlight that men and women have a different epicardial fat deposition and systemic inflammation. Intriguingly, a correlation between visceral/epicardial fat depots and chronic low-grade inflammation was also noted, suggesting that sex may play an essential role in the stratification of obese individuals and dysmetabolic patients.

In conclusion, the experimental data summarized and presented in this Special Issue further strengthen the centrality of lipids in a plethora of biological processes and underline the importance of lipid research in physiopathology. Indeed, lipids may represent useful biomarkers for a number of diseases, and alterations in their metabolism may concur to the development of different disorders. Lipids can also be combined or conjugated to drug compounds, determining several benefits in terms of treatment effect. In this context, further basic, translational, and clinical research are imperative to discover novel mechanisms controlling lipid metabolism in health and disease, and to set up optimal drug design. All these concepts are clearly debated in the concept paper by Markovic et al. [21].
